# The Onchocerciasis Vaccine for Africa—TOVA—Initiative

**DOI:** 10.1371/journal.pntd.0003422

**Published:** 2015-01-29

**Authors:** Peter J. Hotez, Maria Elena Bottazzi, Bin Zhan, Benjamin L. Makepeace, Thomas R. Klei, David Abraham, David W. Taylor, Sara Lustigman

**Affiliations:** 1 Sabin Vaccine Institute and Texas Children’s Hospital Center for Vaccine Development, National School of Tropical Medicine, Baylor College of Medicine, Houston, Texas, United States of America; 2 Institute of Infection and Global Health, University of Liverpool, Liverpool, England; 3 Department of Pathobiological Sciences, School of Veterinary Medicine, Louisiana State University, Baton Rouge, Louisiana, United States of America; 4 Department of Microbiology and Immunology, Sidney Kimmel Medical College, Thomas Jefferson University, Philadelphia, Pennsylvania, United States of America; 5 Division of Pathway Medicine, School for Biomedical Studies, University of Edinburgh, Edinburgh, Scotland; 6 Laboratory of Molecular Parasitology, Lindsley F Kimball Research Institute, New York Blood Center, New York, New York, United States of America; McGill University, CANADA

New supportive health intervention technologies, including a vaccine, may be required in order to achieve onchocerciasis (river blindness) elimination targets. A new transatlantic partnership has been established to develop and test an onchocerciasis vaccine for Africa.

This year marks the 40 year anniversary of a partnership between the World Bank’s Africa Region, the World Health Organization (WHO), Merck & Co., and several other key nongovernmental organizations (NGOs) and agencies who have led global efforts to control onchocerciasis (river blindness) [1]. By almost any measure, the partnership has achieved extraordinary public health successes. It began as the Onchocerciasis Control Programme (OCP) in West Africa, which from 1974 to 2002 initially employed vector control through aerial spraying of insecticides before initiating ivermectin distribution in 1989 [2]. According to WHO, through OCP, tens of millions of onchocerciasis cases were prevented, including an estimated 600,000 cases of blindness [2]. Moreover, OCP successes allowed 100,000 square miles (25 million hectares) of land to be reclaimed for agriculture use [2].

As OCP wound down, a second major initiative, the African Programme for Onchocerciasis Control (APOC) began in 1995, focusing on community-directed mass drug administration (MDA) of ivermectin in 19 countries. According to a recent analysis in *PLOS Neglected Tropical Diseases*, between 1995 and 2010, APOC averted more than 8.2 million disability-adjusted life years (DALYs) and will more than double that number by 2015 [3]. Moreover, the costs of these health benefits are below $500 million, or approximately $27 per DALY averted [3], such that APOC has emerged as one of the world’s most cost-effective global public health programs.

These analyses and evidence that the partnership’s activities led to the elimination of onchocerciasis in endemic foci in two African countries—Mali and Senegal [4]—have fueled great interest and optimism in advancing beyond control and perhaps one day eliminating onchocerciasis from Africa [5]. Indeed, APOC may transition to the Programme for the Elimination of Neglected Diseases in Africa (PENDA) in 2016 with the goal to eliminate onchocerciasis by 2025. This is an ambitious goal that will face a number of challenges.

MDA using ivermectin alone may not be sufficient to achieve onchocerciasis elimination. There are several key barriers. First, a known deficiency of MDA programming is the fact that ivermectin cannot be used in areas coendemic to loiasis due to the risk of severe adverse events (SAEs) [6]. Communities in areas coendemic for both onchocerciasis and loiasis often do not receive treatment [6]. This situation both blocks elimination efforts in Loa-affected communities and also creates reservoir onchocerciasis infections that potentially could promote the reintroduction of onchocerciasis in neighboring communities under MDA treatments [6]. In response, Cameroon has implemented programs of post-treatment surveillance and management in areas where loiasis is coendemic with onchocerciasis, but this practice may not always be feasible elsewhere. In addition, it is common in many areas of sub-Saharan Africa to not implement onchocerciasis MDA programs in areas of hypoendemicity, which could also lead to reintroduction in areas undergoing MDA.

Of even greater concern is the potential widespread emergence of partial or complete drug-resistant *Onchocerca volvulus*, which poses a threat to the long-term effectiveness of using ivermectin alone in all areas [7–9].

Finally, Turner et al. have recently pointed out that success in achieving onchocerciasis elimination would ultimately require irreversible reductions in *O*. *volvulus* microfilariae production by 30–35% following each annual round of ivermectin [9]. However, there is great uncertainty around such estimates [9]. Additional disease modeling studies have suggested that, depending on compliance and levels of parasite transmission, it may not be possible to achieve onchocerciasis elimination even after 50 years of annual ivermectin treatments, thereby necessitating the adoption of biannual treatments, opening the MDA program up to additional logistical and financial challenges [10].

The development and implementation of new tools (such as drugs, diagnostics, and vaccines) may be required if we are to ensure onchocerciasis elimination. Such products could be used to potentiate or enhance the efficiency of ivermectin treatments and address the identified deficiencies of current MDA programming. Examples of such products include the use of moxidectin in MDA programmes inasmuch as its effect in suppressing microfilaria production for prolonged periods [11]; the development of a macrofilaricidal drug or an antibiotic that targets adult parasite bacterial endosymbionts [12]; the development of a new diagnostic for loiasis infection with improved sensitivity and specificity; and the development of a prophylactic and/or therapeutic onchocerciasis vaccine [13].

The Sabin Vaccine Institute Product Development Partnership (Sabin PDP) and academic partners in the United States (New York Blood Center, Thomas Jefferson University, Louisiana State University, Texas Children’s Hospital Center for Vaccine Development at Baylor College of Medicine); Europe (Universities of Edinburgh, Glasgow, Liverpool, Bonn, Imperial College London, and the Natural History Museum of Paris); and Africa (University of Buea, Cameroon, Cameroon Academy of Sciences, and Kwame Nkrumah University, Ghana), have established The Onchocerciasis Vaccine for Africa (TOVA) Initiative, which is pursuing development of an onchocerciasis vaccine (Fig. 1)(S1 Text).

**Figure 1:**
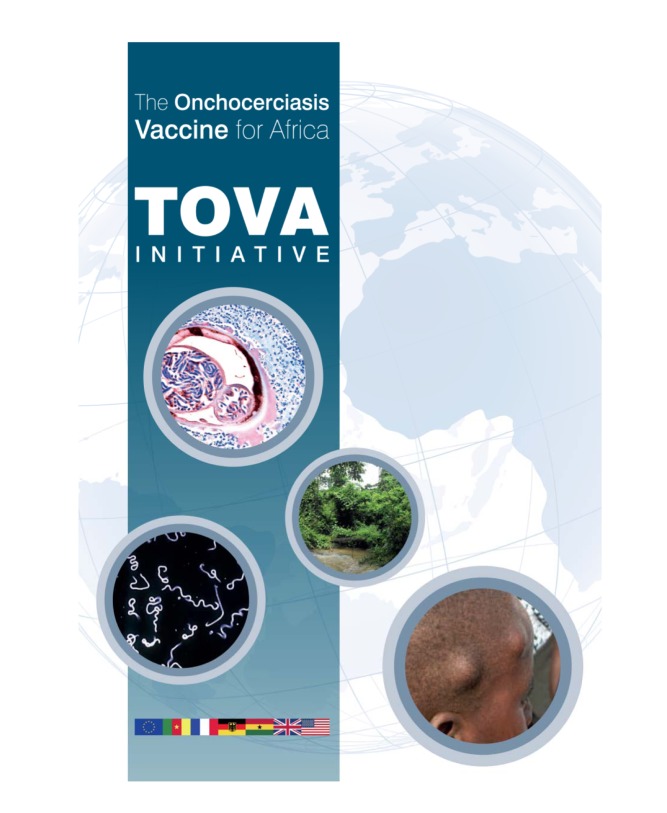
Cover page of The Onchocerciasis Vaccine Initiative prospectus.

TOVA Initiative traces its origins to initial support of more than $20 million during the 1980s and 1990s from the Edna McConnell Clark Foundation, as well as continued support from the European Union to develop molecular reagents, preclinical (laboratory animal) models, understand the effects of infection on the host immunity, and identify key protective antigens across several laboratories in the US, Europe, and Africa. Ultimately, more than a dozen candidate vaccine antigens were identified using small animal models, of which eight were tested in bovines challenged with *Onchocerca ochengi* [14]. Subsequently, these activities continued with the support from NIAID/NIH, together with the European Union (through its Directorate-General for Research and Innovation).

Based on this past research, there are compelling reasons to believe that an onchocerciasis vaccine may be an effective tool. To date, three candidate antigens have proven to be efficacious in three different filarial animal model systems and in three independent laboratories. A recently published study showed feasibility of eliciting significant protective immunity in mice using selected recombinant *Onchocerca volvulus* antigens produced in yeast or bacteria, including *Ov*-103, *Ov*-RAL-2, and *Ov*-CPI-2M [15]. Moreover, the *Brugia malayi* homologous recombinant antigens also elicited protective immunity in gerbils [16] (Klei et al., unpublished). Furthermore, immunization with DNA plasmids carrying the gene encoding *Lg*-CPI-2M protects mice from patent infection with *Litomosoides sigmodontis* [17].

There are at least two potential target product profiles (TPPs) to consider for the development of an onchocerciasis vaccine for Africa. They include the development of a preventive vaccine for use in children five years of age or less, who do not receive ivermectin, or a therapeutic vaccine (targeting either adult worms, microfilariae, the causative agents of pathology and transmission, or both) for children and adults with *O*. *volvulus* infection. These TPPs are not mutually exclusive, and they could potentially also include coadministration with ivermectin or even one of the newer macrofilaricidal agents under parallel development [18]. Modeling studies have shown that an onchocerciasis vaccine could have substantial impact in a range of endemicity settings, and when used as a preventive vaccine, could markedly reduce host microfilarial loads in children and adolescents (Turner HC et al., unpublished).

TOVA Initiative is now establishing a roadmap for developing a vaccine to meet one of the two described TPPs, with plans to take at least one candidate forward to phase two trials (proof-of-concept trial for efficacy) by 2020. Among the key activities envisioned for TOVA Initiative is a program of confirmatory preclinical testing, optimization, and down-selection in the *O*. *ochengi*–cow model under conditions of natural exposure, together with scale-up process development, pilot manufacture, toxicology testing, regulatory filing, and phase one clinical testing. Indeed, TOVA Initiative is poised to lead on the development of this important new tool to aid in the elimination of onchocerciasis.

An onchocerciasis vaccine for Africa would build on past investments in OCP and APOC and support future investments planned under PENDA to help achieve elimination of onchocerciasis [19]. TOVA has begun to explore innovative financing mechanisms from major foundations, governments in North America, Europe, and elsewhere, as well as some of the major development banks committed to poverty reduction in sub-Saharan Africa. We strongly encourage the global public health community to embrace the prospect of an onchocerciasis vaccine and to incorporate plans for a vaccine’s development into future public policy and strategic plan considerations.

## Supporting Information

S1 Text Prospectus for TOVA Initiative. (PDF)
