# Supplementary material for: The Onchocerciasis Vaccine for Africa—TOVA—Initiative
Source: PLoS Negl Trop Dis. 2015 Jan 29;9(1):e0003422. doi: 10.1371/journal.pntd.0003422 (PMC4310604; doi:10.1371/journal.pntd.0003422)
Supplement: S1 Text — (PDF) [file pntd.0003422.s001.pdf]

The **Onchocerciasis Vaccine** for Africa

TOVA

INITIATIVE

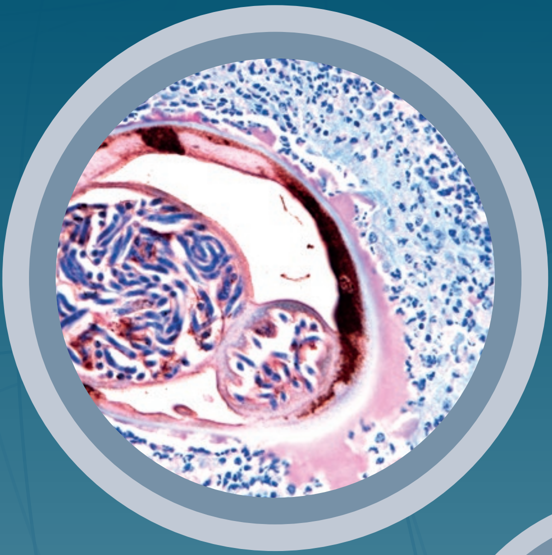

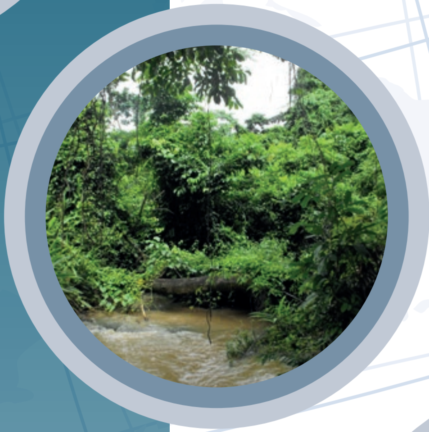

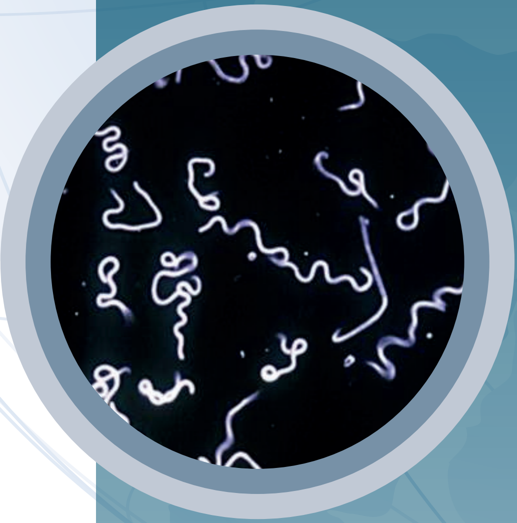

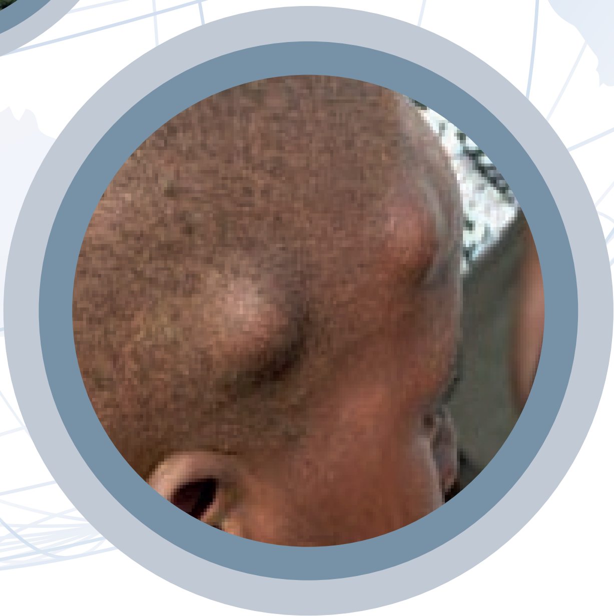

Cover photographs

Section through *Onchocerca* nodule showing microfilariae *in utero*

Blackfly vector breeding site in fast flowing river

Isolated *O volvulus* microfilariae

Onchocerca nodule on child's head

The Onchocerciasis Vaccine for Africa (TOVA) Initiative

New supportive health intervention technologies, including a vaccine, may be required in order to achieve onchocerciasis (river blindness) elimination targets. A new Trans-Atlantic partnership, the Onchocerciasis Vaccine for Africa (TOVA) Initiative, has been established to develop and test an onchocerciasis vaccine for Africa.

From control to elimination through mass drug administration

The London Declaration on Neglected Tropical Diseases of January 2012 called for sustained efforts to expand and extend drug access programmes to ensure the necessary supply of drugs and other interventions to help control human onchocerciasis (river blindness). The African Programme for Onchocerciasis Control (APOC) has extended its mandate, under the new Programme for the Elimination of Neglected Diseases in Africa (PENDA) to begin in 2016, with a new aim of eliminating *Onchocerca volvulus*, the causative agent of onchocerciasis, by 2025.

APOC's work is predicated on sole use of mass drug administration (MDA) of ivermectin (Mectizan™). Moving from control to elimination using MDA of ivermectin alone will be a challenge, and this strategy may not be sufficient to achieve onchocerciasis elimination.

A known deficiency of MDA programming is the fact that ivermectin cannot be used in areas where onchocerciasis and loiasis are co-endemic due to the risk of severe adverse reactions following drug treatment. It is estimated that 12 million people live in such high risk areas in central Africa (Figure 1) and are potentially affected by this contraindication. In these areas, communities often do not receive supportive treatment; onchocerciasis transmission rates remain high; and, reintroduction of the infection to neighbouring communities from which the disease has been eliminated is an ongoing threat.

Figure 1 Onchocerciasis and loiasis high risk areas

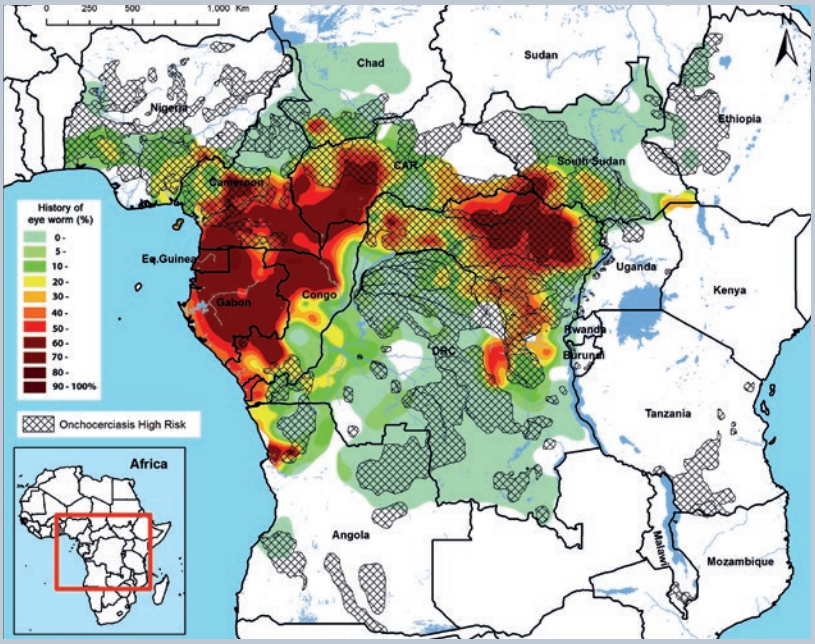

African Programme for Onchocerciasis Control

Additionally, the potential widespread emergence of drug-resistant *O volvulus* poses a threat to the long-term effectiveness of using ivermectin alone in all areas. In some foci, microfilariae are reappearing in the skin following ivermectin treatment at a faster rate than anticipated, and this may be indicative of development of drug resistance.

Successful elimination of onchocerciasis will ultimately require irreversible reductions in *O volvulus* microfilariae production by 30-35% following each annual round of ivermectin treatment. However, there is great uncertainty around such estimates. Disease modelling studies suggest that, depending on compliance and levels of parasite transmission, it may not be possible to achieve onchocerciasis elimination even after 50 years of annual ivermectin treatments, thereby necessitating the adoption of biannual treatments. This would place additional logistical and financial challenges on MDA programmes as well as potentially increasing the rate of emergence of drug resistance. These models recognize the fact that ivermectin does not kill the (long-lived) adult worms and, in areas of high transmission, microfilariae reappear in the skin during the inter-treatment period.

Development of new tools (such as drugs, diagnostics and vaccines) will be required to ensure onchocerciasis elimination and remove the risk of reintroduction of the infection to areas where elimination may have been achieved. Such new tools would potentiate or enhance the efficiency of ivermectin treatments and address the identified deficiencies of the current MDA programming.

The demand

Vaccine as a new tool to aid in elimination

The Onchocerciasis Vaccine for Africa (TOVA) Initiative has been launched as a response to the London Declaration on Neglected Tropical Diseases; and, the scientific and technical demands for new tools to complement ivermectin MDA to ensure elimination of onchocerciasis from Africa.

TOVA has its origins in the river blindness (onchocerciasis) vaccine program of the Edna McConnell Clark Foundation (EMCF) that contributed $21.6 million between 1985 and 1999.

This investment focused on:

- development of experimental animal models for screening candidate vaccine antigens and analysis of mechanisms evoked by immunization with protective recombinant vaccine antigens
- immunological studies in animals and in humans
- identification of protective antigens
- increased understanding of the epidemiology and pathology of river blindness

When the programme ended, the work of African, American and European laboratories had developed three animal models, identified a portfolio of 15 *O volvulus* vaccine candidates including eight that were tested in the *O ochengi* bovine model, and obtained proof-of-principle of vaccination against infection.

The impetus given by EMCF was carried forward by the European Union through its *Directorate-General for Research and Innovation* (FP7, E PIAF, Enhanced Protective Immunity Against Filariasis, coordinated by Professor David W Taylor), and by the US NIH *National Institute of Allergy and Infectious Diseases* (The development of a recombinant vaccine against human onchocerciasis headed by Dr Sara Lustigman).

The work of these programmes (Figure 2) has identified three candidate vaccine antigens that have proven to be efficacious in three different filarial animal model systems and in three independent laboratories (Table 1).

The Onchocerciasis Vaccine for Africa (TOVA) Initiative brings established US and African-European consortia together with the best practice of product development from the Sabin Vaccine Institute and Texas Children's Hospital Center for Vaccine Development, Sabin PDP (Professors Peter Hotez and Maria Elena Bottazzi), and mathematical modelling from Imperial College London (Professor María-Gloria Basáñez).

Figure 2, Progress towards a vaccine against River Blindness

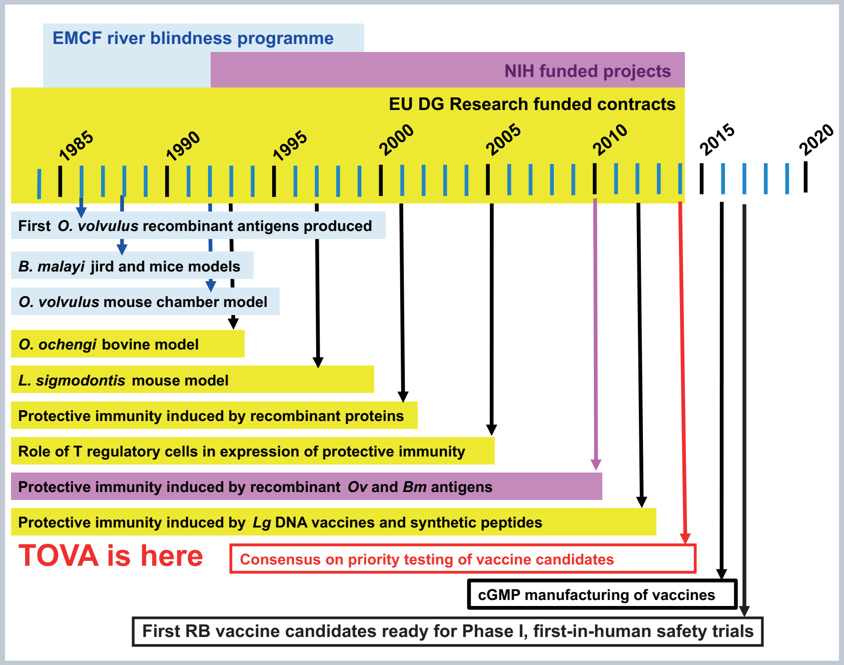

EMCF, Edna McConnell Clark Foundation
NIH, National Institutes for Health USA
EU, European Union

The vaccine

Table 1, Onchocerciasis (river blindness) vaccine candidates

Antigen (expression system)	Location	*In vitro* L3 killing	*In vivo* L3 killing	*In vivo* Adult killing	*In vivo* Microfilaria killing
**CPI-2M** ***(bacteria)***	**ES, Surface, all stages**	***Ov* L3 94%**	***Ov* >30% (rProtein)**	***Ls* 50% (rProtein)**<br>***Bm* >45% (rProtein)**<br>***Ls* 70% (DNA)**	***Ls* >85% (DNA)**<br>***Ls* >85% (synthetic peptide)**
**RAL-2** ***(bacteria)***	**ES, surface, all stages**	***Ov* L3 100%**	***Ov* >40% (rProtein)**	***Bm* >60% (rProtein)**	***Bm* >90% (rProtein)**
**103** **(*yeast*)**	**Surface, all stages**	***Ov* L3 100%**<br>***Ov* Mf >90%**	***Ov* >35% (rProtein)**	***Bm* >40% (rProtein)**	***Ls* >90% (DNA)**

Percentages represent killing *in vitro* (human antigen-specific antibodies + neutrophils) or reduction in parasite burden *in vivo*
ES, excreted-secreted antigens
rProtein, recombinant protein
Ov, Onchocerca volvulus
Bm, Brugia malayi
Ls, Litomosoides sigmodontis
L3, third stage infective larvae
Mf, microfilaria

Protecting children, reducing morbidity and transmission

Our goal is production and testing of a river blindness vaccine to Phase I clinical trials in 2017 and Phase II efficacy trials by 2020.

It is envisaged that the onchocerciasis vaccine will be used initially to protect vulnerable children (<5 years of age) living in loiasis co-endemic areas. The vaccine will reduce adult worm burden and fecundity with consequential reduction in pathology associated with microfilariae (Figure 3). In addition, a vaccine will find use in ongoing ivermectin MDA areas and contribute to reduction in transmission rates; and, will protect areas where local elimination may have been achieved.

Figure 3, The vaccine targets and objectives

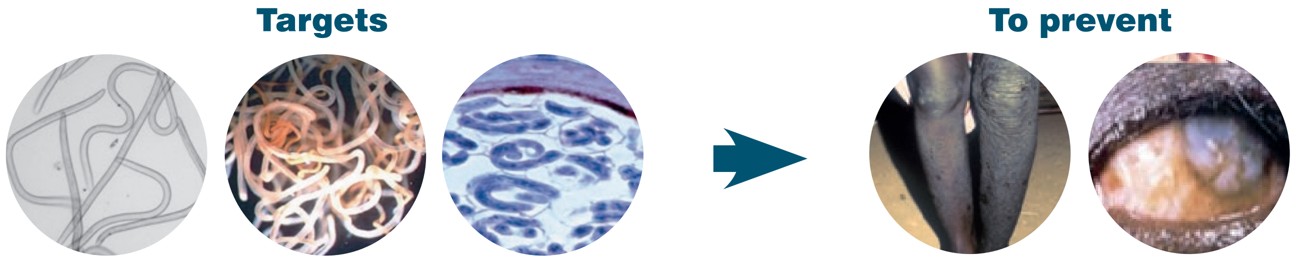

Onchocerca volvulus infective L3 larva — Adult female *O volvulus* from nodule — Microfilariae larvae in uterus

Skin and eye disease

The impact of vaccination

Modelling analyses have shown that an onchocerciasis vaccine will have a substantial impact in a range of endemicity scenarios (Figure 4) and will markedly reduce microfilarial load in those under 20 years of age. This has important implications as studies have highlighted the increased risk of developing onchocerciasis-related morbidity and mortality in individuals who acquire heavy infections in early life.

It is clear that a vaccine would have a beneficial impact by reducing onchocerciasis-related disease burden in these populations. Furthermore, a vaccine could markedly decrease the chance of recrudescence of onchocerciasis in areas where MDA treatment has stopped.

Figure 4, A vaccine will be of greatest benefit to children and young adults

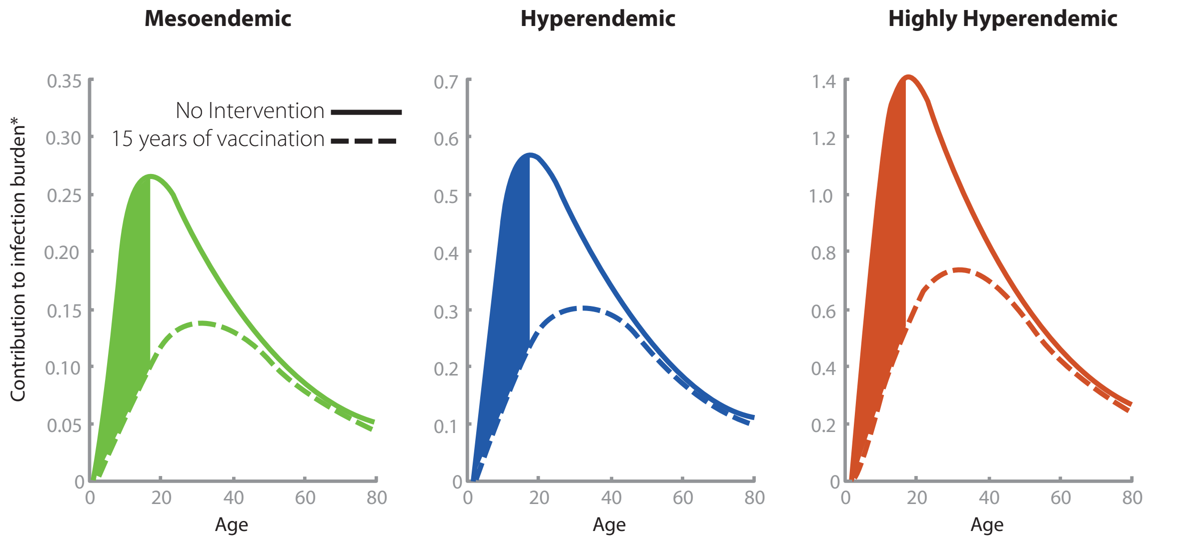

Overall mean mf load decreases by 30% but maximum impact on infection burden in young adults

*age-specific mean mf load multiplied by the probability of being in each age group

These models are based on a vaccination programme that targets children between 1 and 5 years old during its first year, and 1 year old infants during subsequent years. An initial vaccine efficacy of 50% against the infecting L3 larvae and a 90% reduction of skin microfilarial load is assumed giving a mean duration of any protective effects of the vaccine of 20 years with an 80% coverage of vaccination.

Turner et al (unpublished)

Added value

A vaccine would protect the substantial investments made by present and past onchocerciasis control programmes (together, the Onchocerciasis Control Programme in West Africa (OCP) and APOC have cost over US$1 billion), by reducing the chance of disease recrudescence and the possible spread of ivermectin resistance.

What is needed to achieve our mission?

The way forward for vaccine development

TOVA Initiative has set its goal to take at least one vaccine candidate to Phase I trials by 2017 and Phase II trials by 2020. To do this the following tasks have been identified:

1. Mathematical modelling of vaccine efficacy and delivery, as well as cost-effectiveness.
2. Systems analysis to identify specific molecular interactions between parasite antigens and host immune systems to assist with formulation of the vaccine for greatest efficacy and importantly, to avoid any interaction that may lead to adverse reactions, including allergic and physiological responses.
3. Optimization of immunization strategies using the three filarial small animal models.
4. Efficacy trials using the *O ochengi* cattle model under conditions of natural exposure.
5. Process development for human recombinant vaccine production or synthetic vaccines, including formulation, assay development, quality control and stability.
6. Technology transfer for cGMP manufacturing of vaccines.
7. GLP toxicology testing of vaccines.
8. Regulatory filing.
9. Phase I, first-in-human safety trials in developed and in endemic countries.
10. Assessment of immune responses of children up to 9 years age who are exposed to *O volvulus* infections in preparation for phase II trials.

Table 2, Milestones en route to an onchocerciasis vaccine

	Activity	2014	2015	2016	2017	2018	2019	2020
1	Mathematical modelling	●	●	●	●	●	●	●
2	Host and parasite systems analysis for immune correlates and avoidance of pathology	●	●	●	●	●	●	●
3	Optimization of immunization strategies small animal models	●	●	●	●	●	●	●
4	Efficacy trials using the *O ochengi* cattle model		●	●	●	●		
5	Process development for human recombinant vaccine production		●	●	●			
6	Current GMP Manufacturing of vaccines		●	●	●			
7	GLP Toxicology testing			●	●			
8	Regulatory filing			●	●			
9	Phase I first-in-human safety trials				●	●	●	
10	Assessment of immune responses of children	●	●	●	●	●	●	●

Colours identify different work packages. The relative activity of individual work packages are indicated by circle area.

The approach

Table 3, Target product profile of a prophylactic onchocerciasis vaccine

Item	Desired target
Indication	A vaccine to protect against infection with infective (L3) larvae and to reduce adult worm burden and microfiladermia for the purpose of reducing morbidity and transmission.
Target Population	Children $\leq$ 5 years.
Route of Administration	Intramuscular injection.
Product Presentation	Single-dose vials; 0.5 ml volume of delivery.
Dosage Schedule	Maximum of 3 immunizations given 4 weeks apart.
Warnings and Precautions/Pregnancy and Lactation	Mild to moderate local injection site reactions such as erythema, edema and pain, the character, frequency, and severity of which is similar to licensed recombinant protein vaccines. Less than 0.01% risk of urticaria and other systemic allergic reactions. Incidence of serious adverse reactions no more than licensed comparator vaccines.
Expected Efficacy	>50% efficacy at preventing establishment of incoming worms; >90% reduction of microfilariae (based on current animal model results).
Co-administration	All doses may be co-administered and/or used with other infant immunization programmes.
Shelf-Life	4 Years.
Storage	Refrigeration between 2 to 8 degrees Celsius. Cannot be frozen. Can be out of refrigeration (at temperatures up to 25 degrees) for up to 72 hours.
Product Registration	Licensure by the Food and Drug Administration and/or the European Medicine Agency.
Target price	Less than $10 per dose for use in low- and middle-income countries.

TOVA: who we are

TOVA Initiative represents a collaborative effort between a team of experienced investigators who have been working together on river blindness for 30 years. These investigators are supported by young scientists with expertise ranging from mathematical modelling, through immunology, proteomics and genomics, vaccinology and product development to clinical practice. TOVA Initiative will adopt the Product Development Partnership (PDP) approach used by the Sabin PDP for accelerating the development of new vaccines for global health.

Table 4, TOVA Partners

Name	Participant's organization, country	Role in the Partnership
**The partners from Africa**		
Professor Samuel Wanji	University of Buea, Cameroon<br>Research Foundation in Tropical Disease and Environment	Human studies in Cameroon
Dr Vincent Tanya	Cameroon Academy of Sciences	Screening vaccine candidates in the *O ochengi* cow model
Dr Alex Debrah	Kwame Nkrumah University, Ghana	Human studies in Ghana
**The partners from Europe**		
Professor David W Taylor	University of Edinburgh, UK	Co-ordinator of the EU consortium.<br>Host gene expression profile analysis.<br>Vaccine development and human studies in Cameroon
Dr Ben Makepeace<br>Professor Jonathan Wastling	University of Liverpool, UK	Proteomic and genomic analyses and vaccine development. Screening vaccine candidates in the *O ochengi* cattle model
Dr Simon Babayan	University of Glasgow, UK	Filarial immunology, vaccine development and screening vaccine candidates in the *L sigmodontis* mouse model
Dr Coralie Martin	Muséum National d'Histoire Naturelle, Paris, France	Screening vaccine candidates in the *L sigmodontis* mouse model. Host gene expression profile analysis
Professor Achim Hoerauf<br>Dr Sabine Specht	University Hospital of Bonn, Germany	Immunology of filarial infections and human studies in Ghana.<br>Host gene expression profile analysis
Professor María Gloria Basáñez	Imperial College London, UK	Mathematical modelling and cost-effectiveness
**The partners from USA**		
Dr Sara Lustigman	New York Blood Center, NYC, USA	Program Director of the NIH funded consortium. Human studies in Cameroon, characterization of vaccine candidates
Professor David Abraham	Thomas Jefferson University, Philadelphia, PA, USA	Screening vaccine candidates in the *O volvulus* mouse model
Professor Thomas Klei	Louisiana State University, Baton Rouge, LA, USA	Screening vaccine candidates in the *B malayi*-jird model
Professor Maria Elena Bottazi<br>Professor Peter Hotez	The Sabin Vaccine Institute and Texas Children's Hospital Center for Vaccine Development, Houston, TX, USA	Product development, technology transfer for cGMP manufacture and GLP toxicology testing, regulatory filing, early stage clinical testing

TOVA is an unincorporated affiliation of the institutions listed in Table 4 that are working collaboratively to meet the challenges of developing a vaccine against river blindness.

TOVA: where we are

REFOTDE

University of Buea / Université de Buea — Knowledge with Wisdom

Cameroon Academy of Sciences / Académie des Sciences du Cameroun

KWAME NKRUMAH UNIVERSITY OF SCIENCE AND TECHNOLOGY

KCCR Kumasi Centre for Collaborative Research in Tropical Medicine

New York Blood Center Lindsley F. Kimball Research Institute

Jefferson. Thomas Jefferson University

Baylor College of Medicine National School of Tropical Medicine

Texas Children's Hospital

SABIN VACCINE INSTITUTE

LSU School of Veterinary Medicine

Imperial College London

universitätsklinikumbonn

MUSÉUM NATIONAL D'HISTOIRE NATURELLE

THE UNIVERSITY of EDINBURGH

University of Glasgow

UNIVERSITY OF LIVERPOOL

Funding Acknowledgements:

EU FP5, VARBO, ICA-CT-1999-10002

EU FP6, SCOOTT, INCO 032321

EU FP7, E PIAF, 131242

NIH/NIAID R01AI078314

NIH/NIAID R01A1423228

SEVENTH FRAMEWORK PROGRAMME

The **Onchocerciasis Vaccine** for Africa

TOVA INITIATIVE

For further details contact:

Professor David W Taylor
School for Biomedical Studies
University of Edinburgh
David.W.Taylor@ed.ac.uk

Dr Ben Makepeace
Institute of Infection & Global Health
University of Liverpool
blm1@Liverpool.ac.uk

Dr Sara Lustigman
Lindsley F Kimball Research Institute
New York Blood Center
SLustigman@nybloodcenter.org
